# Dynamic expression of chromatin modifiers during developmental transitions in mouse preimplantation embryos

**DOI:** 10.1038/srep14347

**Published:** 2015-09-25

**Authors:** Peter Nestorov, Hans-Rudolf Hotz, Zichuan Liu, Antoine H.F.M. Peters

**Affiliations:** 1 Friedrich Miescher Institute for Biomedical Research, 4058 Basel, Switzerland; 2 Faculty of Sciences, University of Basel, 4056 Basel, Switzerland

## Abstract

During mouse preimplantation development, major changes in cell fate are accompanied by extensive alterations of gene expression programs. Embryos first transition from a maternal to zygotic program and subsequently specify the pluripotent and the trophectodermal cell lineages. These processes are regulated by key transcription factors, likely in cooperation with chromatin modifiers that control histone and DNA methylation. To characterize the spatiotemporal expression of chromatin modifiers in relation to developmental transitions, we performed gene expression profiling of 156 genes in individual oocytes and single blastomeres of developing mouse embryos until the blastocyst stage. More than half of the chromatin modifiers displayed either maternal or zygotic expression. We also detected lineage-specific expression of several modifiers, including *Ezh1, Prdm14, Scmh1* and *Tet1* underscoring possible roles in cell fate decisions. Members of the SET-domain containing *SMYD* family showed differential gene expression during preimplantation development. We further observed co-expression of genes with opposing biochemical activities, such as histone methyltransferases and demethylases, suggesting the existence of a dynamic chromatin steady-state during preimplantation development.

The first steps of mouse embryogenesis involve extensive chromatin remodelling of the two parental genomes, followed by activation of zygotic transcription and two subsequent cell fate specifications^1^. These critical processes take place in a narrow time window of four days and are substantially controlled by maternally provided components^2^. Initiation of global transcription is known as zygotic genome activation (ZGA) and occurs in multiple successive waves. ZGA starts in the zygote stage and continues with major bursts of *de novo* transcription at the 2- and 4-cell stages, while at the same time the maternally provided transcripts are degraded^3^,^4^.

The first two lineages in mouse embryos are the trophectoderm and pluripotent cells of the early blastocyst (32-cell stage), representing the outer layer and the inner cell mass (ICM) respectively. Lineage specification is regulated by transcriptional networks, driven by lineage-specific transcription factors, such as Cdx2, Sox2 and Pou5f1 (alias Oct4). The first significant differences between inner and outer cells in terms of cell fate and gene expression become evident at the 16-cell stage^5^, but a number of studies suggest that cell fate specification is initiated well before the 16-cell stage^6^,^7^. It is not yet fully understood which mechanisms specify cell fate in a system with high cell plasticity and stochastic fluctuations.

Before the first cell lineages are specified, there is extensive chromatin remodelling accompanied by alterations in histone and DNA methylation. The most striking changes occur in the zygote, where the two parental genomes are organized in two separate pronuclei. The paternal genome is inherited in a highly compacted and a largely histone-depleted conformation, and undergoes rapid decondensation and incorporation of maternal histones and protein complexes shortly after fertilization^8^. Subsequently the paternal pronucleus gains mono-, di- and tri-methylation on lysines 4 of histone H3 (H3K4me1, H3K4me2 and H3K4me3) and likewise on lysine 27 of H3 while these marks are inherited from the oocyte and maintained in the female pronucleus. The DNA methylation state is also actively reprogrammed by Tet3 proteins as well as passively via replication-associated mechanisms^1^,^9^,^10^. Interestingly, di-methylation of H3 on lysine 9 (H3K9me2) has been suggested to protect against DNA demethylation through the activity of Dppa3 (also known as PGC7/Stella), which binds to H3K9me2 and shelters the maternal genome from hydroxymethylation by the Tet3 protein^11^. It has further been proposed that chromatin-borne parental information, recognized by chromatin-binding proteins, may influence ZGA and lineage specification in the mouse embryo^12^,^13^,^14^. Thus, the dynamic changes in chromatin states that take place in a parent-of-origin specific manner during preimplantation development suggest important functions for histone modifying enzymes during early embryonic development.

Genome-wide expression profiling on pooled embryos gives global insights into the expression changes that occur during development^15^,^16^,^17^. However, given the partially stochastic nature of the developmental processes taking place in preimplantation embryos^6^,^7^,^18^,^19^, single-cell profiling approaches have a higher potential to shed light on the role of transcription factors and chromatin modifiers in defining gene expression states. Recent genome-wide RNA sequencing efforts have revealed complex expression patterns of moderately to highly expressed genes in single embryos^20^,^21^. The Biomark HD system (Fluidigm Corp., San Francisco, USA) and the dedicated microfluidic arrays enable an alternative, highly sensitive, measure for quantitative expression analyses of several hundred selected genes in single cells. The later method has been first used in mouse embryos by Guo and colleagues for a set of 48 genes^5^. While Guo *et al.* focused on certain lineage-specific genes, we describe here the expression dynamics of over 150 genes encoding for chromatin modifying proteins in preimplantation embryos at a single-cell resolution.

## Results

### Selection of genes and single-cell profiling

**Figure 1:**
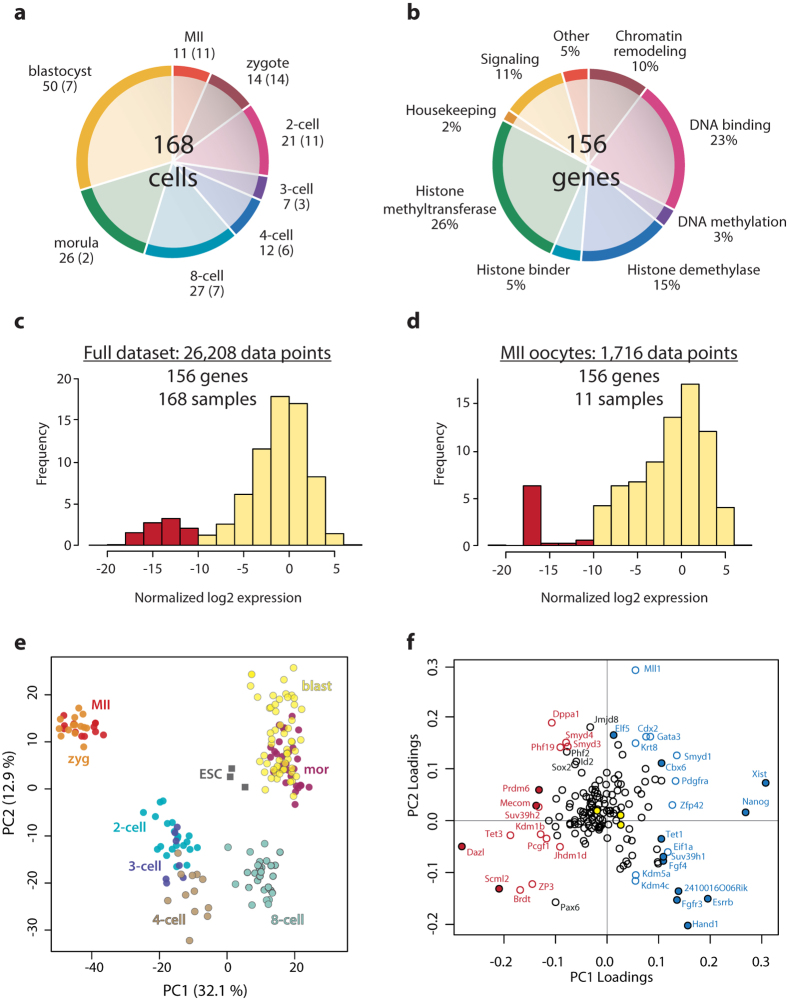
Single-cell expression of chromatin modifiers during preimplantation development. (**a**), Distribution of oocytes, zygotes and blastomeres according to stage. Numbers of embryos are shown in brackets. (**b**), Distribution of genes by suggested function. Since some genes are suggested to have multiple functions, the distributions are given as a percentage (some genes were attributed to more than one function). **c,** Frequency distribution of normalized log2 expression signal for the complete dataset. Red bars indicate samples below the defined threshold of detection of −10. (**d**), Frequency distribution of normalized log2 expression values for the MII oocyte samples only (maternal expression). Red bars indicate samples below the defined global threshold of detection of −10. (**e**), Principal component analysis (PCA) for complete dataset. Data points correspond to samples (single cells) and are coloured by embryonic stage. In addition, three samples from embryonic stem cells RNA (ESC) are included in the PCA. Percentages indicate the variability explained by the first and second principal component respectively. (**f**), Loadings plot for the PCA shown in Fig. 1e. The further away from the origin of the graph, the stronger it contributes to variability between the samples. Genes are colour-labelled based on their expression pattern. Filled red data points correspond to exclusively maternal genes, filled blue data points correspond to exclusively zygotic genes. Red circles correspond to predominantly maternal genes, blue circles correspond to predominantly zygotic transcript (showing expression in all stages, but with a clear trend towards maternal or zygotic expression respectively). Yellow data points indicate the three reference genes *Hnrnpr*, *Ssu72* and *Ube2e1*.

In order to understand how chromatin modifying complexes take part in the dynamic transcriptional changes during mouse preimplantation development, we selected 192 genes based on their function and assayed their expression in a total of 168 oocytes, zygotes and single blastomeres and a few groups of blastomeres isolated from preimplantation embryos (Fig. 1a, Supp. Table 1). For qPCR detection on the BioMark 96.96 chips, we used EvaGreen chemistry which enables the monitoring of unspecific PCR products and primer dimers. After performing quality control analysis using the BioMark software, we discarded data for 36 genes due to non-specific signal. We next performed in depth data analysis for 156 remaining genes encoding for histone methyltransferases, histone demethylases, chromatin-binding proteins, as well as key transcription factors and signalling molecules. As a reference set for stage- and lineage-specific expression, we included 18 of the 48 genes used in the first Fluidigm-based study of mouse embryos by Guo *et al.*^5^. We converted raw Ct values to expression values and normalized them relative to the mean expression signal of three endogenous control genes *Hnrnpr*, *Ssu72* and *Ube2e1* (Fig. 1b, Supp. Table 1, GEO accession: GSE63632).

### Expression dynamics during the maternal-to-zygotic transition

Based on the frequency distribution of all normalized expression values, we defined the threshold of expression to be -10 (Fig. 1c). Using this threshold, we identified that the majority of the 156 analysed genes were expressed throughout preimplantation development. There were only 16 genes showing exclusive zygotic expression (mean signal in MII oocytes below −10), another 7 genes were exclusively maternal (expressed above threshold in MII oocytes but not in morula and blastocyst cells) and 2 genes were not expressed at all (Fig. 1d, Supp. Table 2). The dynamic expression of the selected genes allowed us to confidently resolve the developmental transitions that occur during preimplantation development, which is visualized in the principal component graph in Fig. 1e. The strongest difference was the transition from the transcriptionally silent MII and permissive zygote stages to the transcriptionally active 2-cell and later preimplantation stages, i.e. the maternal-to-zygotic transition. The relative contribution of single genes to the two principal components is visualized in Fig. 1f (see also Supp. Table 2). The genes that account for most of the variation between single cells (i.e. genes that have highest loading value for the principal component analysis in the first two projections) are the maternal genes *Dazl, Mecom* (alias *Prdm3, Evi1*)*, Prdm6 and Scml2* on one hand, and the zygotically expressed genes *Cbx6, Elf5, Esrrb, Fgf4, Fgfr3, Hand1, Nanog, Suv39h1, Tet1, Xist* and *2410016O06Rik* (alias *NO66*) on the other. Furthermore, another group of genes that are continuously expressed from oocyte through blastocyst exhibited nonetheless clear changes in expression levels between stages and therefore contributed significantly to the first two principal components. This group comprises the predominantly maternal genes *Brdt, Dppa1, Jhdm1d, Kdm1b, Pcgf1, Phf19, Smyd3, Smyd4*, *Suv39h2, Tet3* and *Zp3* and the predominantly zygotic genes *Cdx2, Eif1a, Gata3, Kdm4c, Kdm5a, Krt8, Mll1, Pdgfra, Smyd1* and *Zfp42*.

**Figure 2:**
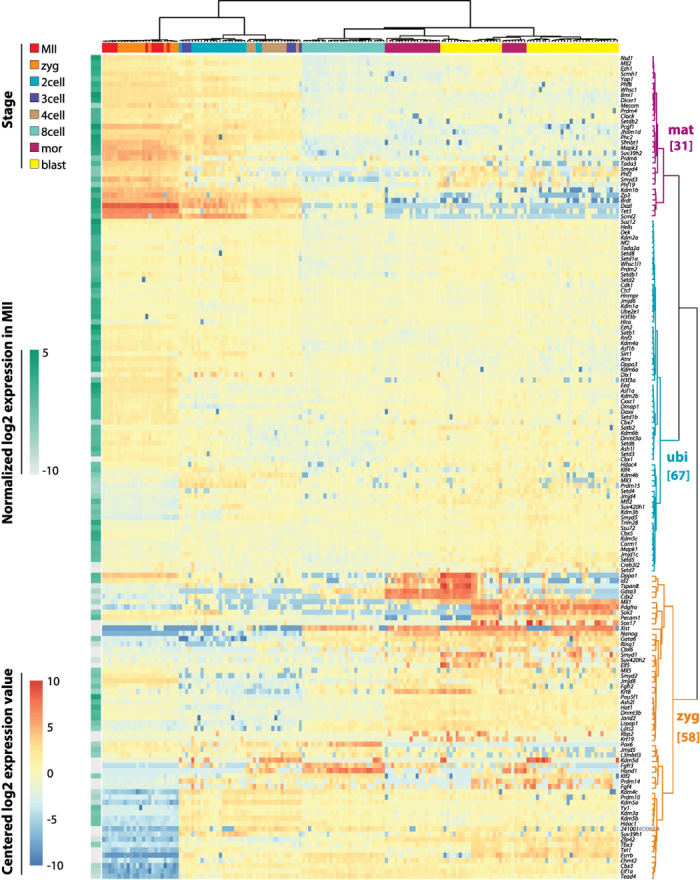
Expression patterns during preimplantation development. Heatmap of the normalized log2 expression data, centred by subtraction of the mean on a by-gene basis. The absolute expression level in MII oocytes is represented by the green heatmap column next to the main heatmap, serving as a reference for the maternal load of each gene. Hierarchical clustering highlighted three main branches, corresponding to “maternal”, “ubiquitous” and “zygotic” expression patterns.

Next, we looked for genes with similar expression patterns throughout preimplantation development, regardless of their absolute expression level. We centred expression values for each gene (subtraction of mean) and applied hierarchical clustering to the centred dataset (Fig. 2). The clustering gave three main branches, which we broadly classified as “maternal”, “ubiquitous” and “zygotic”, respectively, corresponding to a decreasing, constant, or increasing expression from MII oocyte to blastocyst stage. Most of the analysed genes (n = 67) demonstrated stable expression levels across stages. Slightly less genes (n = 58) were expressed stronger upon zygotic genome activation at the 2-cell stage. Finally, 31 genes exhibited constantly decreasing levels, suggesting maternally provided transcripts subject to degradation. We note that the “zygotic” cluster consists of several subsets of genes that show differential expression dynamics. Such stage specific expression dynamics is in line with previously reported transcriptional changes as measured by micro-array approaches^17^,^22^. In particular, some of the “zygotic” genes showed also maternal expression, however weaker than the expression detected in later stages. Also, the “zygotic” cluster includes most of the lineage-specific genes, which are expressed only in a subset of the 16-cell and 32-cell stage and form a separate subcluster. In this respect, some “maternal” cluster genes also showed differential expression in the morula and blastocyst embryos, though not necessarily correlated to the inner/outer lineage-specific cell identity.

### Lineage-specific expression

**Figure 3:**
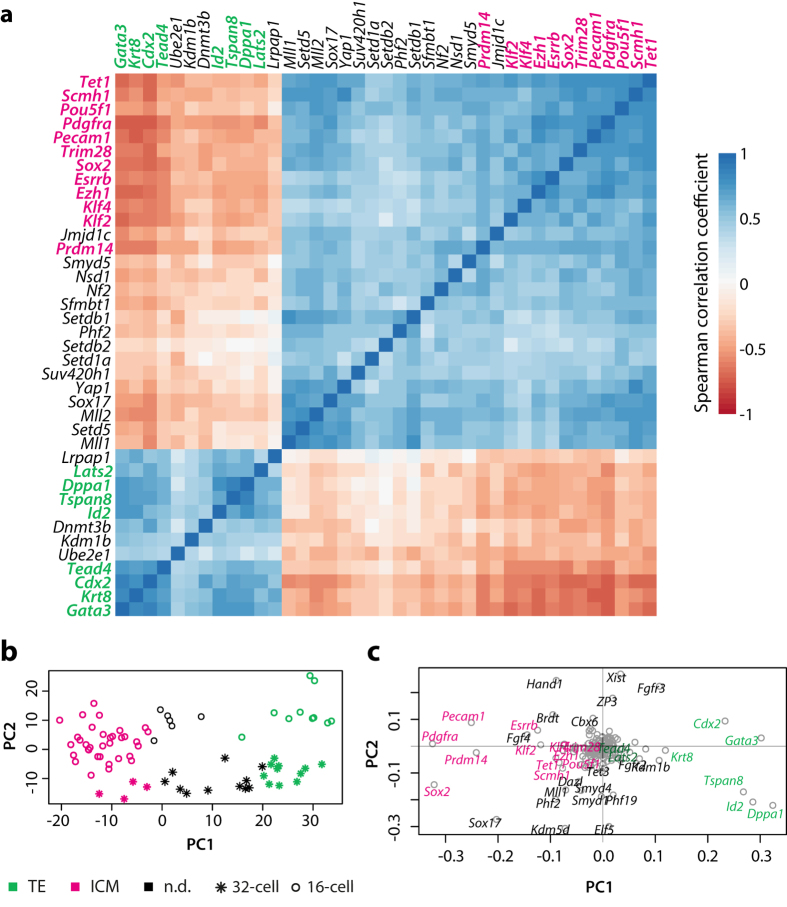
Lineage specific gene expression. (**a**), The expression of all genes in the 16-cell and 32-cell blastomeres was used to generate the clustered correlation matrix shown in Supp. Fig. 1a. Here, only the two anti-correlating clusters are given. Genes that were defined as “core lineage markers” are highlighted in purple (pluripotency, ICM) or green (trophectoderm, TE). (**b**), PCA plot for blastomeres of 16- and 32-cell embryos, coloured according to the lineage score, calculated from the expression of the highlighted genes in panel 3a (see also Supp. Table 3). The shape of the points indicates the stage, while the colour corresponds to the assigned cell fate. **c**, Loadings plot for the core lineage markers showing their relative contribution to the PCA in 3b.

After we identified a significant change during the maternal-to-zygotic transition, we asked whether there is also a difference in the expression of chromatin modifiers along with the first cell fate specification events. As evident in the global PCA graph (Fig. 1e), there is a dramatic shift in gene expression from 8-cell to morula and blastocyst stage blastomeres. To analyse this issue in more detail, we generated a correlation matrix for the expression of 156 genes in 16-cell and 32-cell blastomeres and performed hierarchical clustering (Fig. 3a, Supp. Fig. 1a). Next we determined the statistical significance of the correlations and clustered the genes according to the corresponding p-values (Supp. Fig. 1b, Supp. Tables 4 and 5). In both analyses, we identified two clusters of genes with mutually-exclusive expression patterns, representing “ICM” and “trophectoderm”-related gene sets. As part of the core ICM set, we confirmed the presence of several transcription factors such as *Pou5f1, Sox2, Klf2, Klf4,* and signalling related molecules like *Esrrb, Pecam1* and *Pdgfra* that had been previously identified by Robson and colleagues^5^. Expression of several chromatin modifiers such as the Polycomb-genes *Ezh1* and *Scmh1*, the co-repressor *Trim 28* (*Kap1 or TIF1 *β**), the DNA demethylation enzyme *Tet1* and the histone methyltransferase *Prdm14* was strongly correlated to the known ICM markers^23^. In addition, expression of several other histone methyltransferases like *Nsd1, Setdb1, Setdb2, Setd1a, Setd5, Smyd5* and *Suv420h1* and the Trithorax-group genes *Mll1* and *Mll2* (alias *Kmt2a* and *Kmt2b*) as well as the H3K9-specific histone demethylases *Jmjd1c* and *Phf2,* the Polycomb protein *Sfmbt1* and the transcription factor *Tbx3* correlated with those of core ICM genes as well.

As part of the trophectoderm-related gene set, we observed known markers like *Cdx2*, *Dppa1, Gata3, Id2*, *Krt8, Tead4 and Tspan8*. We identified a moderate trophectoderm-like expression pattern for the DNA methyltransferase *Dnmt3b* and the H3K4-specific histone demethylase *Kdm1b*. Notably, expression of two components of Hippo signalling were inversely correlated with cell fate. While *Lats2* expression correlated with trophectoderm-related genes, *Yap1* correlated with ICM markers.

We next aimed at estimating the capacity by which the different groups of genes can predict the lineage fate of individual blastomeres. Combining a lineage scoring algorithm with a principal component analysis, we were able to classify most blastomeres according to expression of the core ICM and trophectoderm gene sets (Fig. 3b). As expected, the two sets of anti-correlating ICM- and TE-specific genes were accounting for most of the variability along the first principal component, which captures the lineage differences (Fig. 3c, Supp. Table 3). Notably, we were able to faithfully assign the cell fates of the blastomeres solely based on the signal of the four genes *Ezh1, Prdm14, Scmh1* and *Tet1* (Supp. Fig. 2), underscoring the lineage specific expression and possible function of these epigenetic factors, as shown before for *Prdm14* and *Tet1*^23^,^24^,^25^.

In summary, these analyses broaden our understanding of the lineage-specific expression changes that accompany the first cell fate decisions by identifying a set of epigenetic modifiers that follow the expression of transcription factors and signalling molecules.

### Expression of chromatin modifying complexes

**Figure 4:**
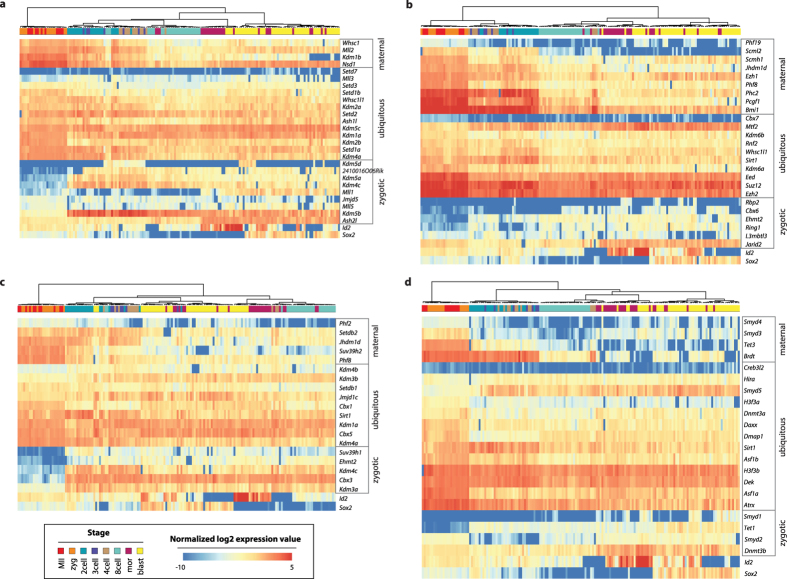
Expression dynamics of epigenetic pathways during preimplantation development. Heatmaps showing the normalized log2 expression values of genes coding for proteins that are associated with H3K4 and H3K36 methylation (**a**), H3K27 methylation (**b**), H3K9 methylation (**c**) and DNA methylation, histone variants, histone chaperones and SMYD family genes (**d**). The representation of the normalized log2 expression values allows comparison of expression intensities between genes. The annotation of the genes as “maternal, “ubiquitous” or “zygotic” corresponds to the clusters shown in Fig. 2. As a reference, the expression dynamics of the lineage-specific genes Id2 and Sox2 is also depicted.

Following the global analysis, we decided to zoom in on specific protein complexes and chromatin modification pathways. For several protein families we observed that paralogous genes display either a maternal or zygotic expression pattern, suggesting stage specific functions (Fig. 4). Notable examples are the predominantly maternal H3K4 histone methyltransferase *Mll2* versus its zygotic homolog *Mll1*^26^ (Fig. 4a), as well as the maternal H3K9 methyltransferase *Suv39h2* versus the zygotic *Suv39h1*^27^ (Fig. 4c). In contrast, the core members of Polycomb repressive complex 2 (PRC2) (*Ezh2, Eed* and *Suz12*) are ubiquitously and highly expressed throughout preimplantation development^28^ (Fig. 4b). Core members of PRC1, the other major Polycomb repressive complex, show more diversity with *Bmi1* being maternal and *Ring1* and *Cbx6* being strongly zygotic^29^. Interestingly, most of the histone demethylases are predominantly zygotically expressed.

Finally, we also analysed in detail the DNA methylation and chromatin remodelling players, as well as the poorly studied SMYD family of histone methyltransferase genes (Fig. 4d). We observed that *Dnmt3a* and *Dnmt3b*, two enzymes required for *de novo* DNA methylation, were predominantly maternally and zygotically expressed, respectively. This observation is consistent with protein levels of the two enzymes detected by immunofluorescence in preimplantation embryos^30^. We also measured a maternal versus zygotic division of labour for *Tet3* and *Tet1*, respectively, two homologous enzymes that oxidize methylated cytosine (5-mC) to hydroxymethyl-cytosine (5-hmC).

All of the screened histone chaperones and chromatin remodellers were ubiquitously expressed from oocyte to blastocyst. As for the SMYD family genes, we again observed maternal-zygotic differences between the family members – *Smyd3* and *Smyd4* were enriched in the maternally-controlled stages, while *Smyd1*, *Smyd2* and *Smyd5* were expressed more strongly after ZGA. Notably, *Smyd1* displayed exclusively zygotic expression starting from the 8-cell stage on. The heterogeneous expression of *Smyd1* did not correlate with the lineage markers. In contrast, *Smyd5* showed higher expression in the ICM cells (Fig. 3a).

## Discussion

By using specific target amplification coupled to microfluidic PCR, we quantified the expression of 156 genes in single-cell resolution during the first stages of mouse embryogenesis. These analyses revealed the expression dynamics of single genes during preimplantation development in relation to the other 155 genes. We confirmed previously shown transcriptional dynamics during preimplantation development of several transcription and signalling factors and a limited set of chromatin modifiers^5^,^23^ and significantly extended the depth of the single-cell profiling by including three times more targets with an emphasis on genes encoding chromatin modifiers.

We identified many transcripts showing a maternal- or zygotic-specific expression pattern. Intriguingly, paralogs of several chromatin modifying enzymes showed either pronounced maternal or zygotic expression, suggesting developmentally controlled division of labour. Examples are the major H3K9 methyltransferases *Suv39h1* and *Suv39h2*^27^ and the DNA methyl-cytosine dioxygenases *Tet1* and *Tet3*^31^. We also observed that the zygotic paralog *Tet1* is differentially expressed upon lineage specification and is enriched in the inner cell mass, which is in line with the described function of *Tet1* in maintaining the pluripotent state by preventing methylation of the Nanog promoter^25^. We provide data for the poorly characterized genes of the SMYD family and identified *Smyd2, Smyd3, Smyd4* and *Smyd5* as maternally provided, while *Smyd1* is expressed only after ZGA. *SMYD* genes have been shown to regulate transcription during muscle development by H3K4/H3K36 methylation but their role in germ cells and early embryos remains largely unexplored^32^,^33^,^34^.

An interesting and not yet fully understood issue in chromatin-based gene regulation is the co-expression of methyltransferases and demethylases modifying the same histone residues. The presence of both the “writer” and the “eraser” suggests a highly dynamic methylation state, maintained by the balance between the two opposing enzymes^35^. Our study included a total of 24 histone demethylases and 41 histone methyltransferases, the majority of which were co-expressed during preimplantation development. Two demethylases showed lineage-specific expression in late preimplantation embryos. The H3K4-specific histone demethylase *Kdm1b* is strongly maternal but remains expressed also after ZGA and eventually becomes excluded from many of the ICM cells. The non-ubiquitous expression pattern and correlation with trophectoderm markers makes *Kdm1b* a good candidate for lineage-specification studies. Another protein with a potential function in lineage specification is the putative^36^ demethylase *Jmjd1c*, which is enriched in ICM blastomeres of 16- and 32-cell embryo. In summary, we reveal the maternal-zygotic dichotomy within major epigenetic complexes and pathways, as well as the lineage-specific expression of some key chromatin modifying genes.

## Methods

**Oocyte and embryo collection, *in vitro* culture and single cell isolation.** F1 female mice (C57BL/6 x DBA/2) were superovulated and in the case of embryo collection, mated to C57BL6 males. MII oocytes were collected 14 hours post hCG injection in M2 medium (Sigma, M7167). Zygotes were collected 14–16 hours post hCG collection in M2 medium. Cumulus cells were removed by incubation for 5 min in 1 mg/ml Hyaluronidase-containing M2. Embryos were cultured in KSOMaa (Milipore, MR-106-D) in a low oxygen air chamber. Single blastomeres were isolated by first removing the *Zona pellucida* with Proteinase (Sigma, P5147-1 G, 5 mg/ml in M2 medium), followed by separating the cells with Trypsine (Trypsine-EDTA, T4049 Sigma). Single cells were picked with the help of a mouth pipette and a finely pulled glass capillary. All experiments were performed in accordance with the Swiss Animal Protection laws and institutional guidelines and were approved by Cantonal Veterinary Office (Basel-City, Switzerland).

### Preparation of pre-amplified single-cell cDNA

Specific target amplification was performed by pipetting single cells directly into 0,1 ml nuclease-free PCR tubes containing the CellsDirect™ One-Step qRT-PCR Kit (Invitrogen, 11753-100) reaction mix . Each reaction amounted 4.5 μl and contained 1.25 μl primers mix (192 forward and 192 reverse primers mixed at a final concentration of 500 nM each), 2.5 μl 2x CellsDirect master mix, 0.1 μl CellsDirect Enzyme mix, 0.1 ul Superase-In (Ambion, AM2696) and 0.55 μl DNA Resuspension Buffer (TEKnova, T0221). Reverse transcription was performed by incubation in a standard thermal cycler for 15 minutes at 50 °C, followed by 2 minutes at 95 °C and subsequently 18 cycles of PCR amplification (15 seconds at 95 °C and 4 minutes at 60 °C).

Residual primers were removed by adding 2 μl Exonuclease I master mix to the pre-amplified reactions. The Exonuclease I mastermix consisted of 0.4 μl Exonuclease I (New England BioLabs, M0293S), 0.2 μl 10x Reaction buffer (provided with enzyme) and 1.4 μl nuclease-free water. The samples were incubated for 30 min at 37 °C, followed by 15 min at 80 °C. The pre-amplified reaction products were diluted 5-fold and stored at −20 °C.

### Single-cell qPCR with BioMark 96.96 GE Dynamic Arrays

Sample Pre-Mix solutions were prepared in two 96-well plates for the 192 samples, using 2.7 μl of the pre-amplified cDNA, mixed with 3.0 μl 2X Sso Fast EvaGreen Supermix With Low ROX (Bio-Rad Laboratories, PN 172-5211) and with 0.3 μl 20X DNA Binding Dye Sample Loading Reagent (Fluidigm, PN 100-0388). The assay mix for the 192 assays was prepared by mixing 3.0 μl 2X Assay Loading Reagent (Fluidigm, PN 85000736) with 2.7 μl 1X DNA Suspension Buffer (Teknova, PN T0221) and 0.3 μl 100 μM each of Forward and Reverse Primer Mix. Four 96.96 GE Dynamic Array (Fluidigm, PN BMK-M-96.96) chips were loaded and run on a BioMark system as described by the manufacturer (Protocol ADP 41, Fluidigm).

### Data analysis and visualization

Data from the BioMark qPCR system was processed with Fluidigm Real-Time PCR Analysis Software, including quality control of the experiment and identification of unspecific products based on the product melting temperature. The Ct values obtained from the BioMark System were converted into relative expression levels by subtracting the values from the assumed baseline value of 30 (inverted Ct values). Cells with low or absent endogenous control gene expression levels were removed from analysis. We also removed genes with no specific signal from the analysis. The resulting values (156 genes and 189 samples) were normalized to the average signal for the endogenous reference genes *Ssu72, Hnrnpr* and *Ube2e1*, by subtracting the average inverted Ct value for the three reference genes from the respective expression value. The normalized expression values were analysed and visualised in R.

Correlation analysis of gene expression in the 16-cell and 32-cell stage was done with GraphPad Prism 6, by computing the nonparametric Spearman correlation and the respective p-values (two-tailed, confidence interval 95%), pairwise for all 156 genes. The correlation and p-value matrices were plotted in R using hierarchical clustering. Core lineage-specific genes were defined based on the clustering observed in the correlation and p-value matrices, ultimately giving two sets of ICM- and TE-specific genes respectively. We performed principal component analysis on the subset of 16- and 32-cell blastomeres samples and tested the relative contribution of each of the lineage-specific sets of genes when assigning a cell fate to a given sample. To do so, we took the average expression value of a given gene set, defined thresholds for low, mid and high expression and based on this, we assigned each sample as being ICM, TE or not defined (n.d.). In Fig 3c, we calculated a “Lineage score”, by taking the average expression value of ICM-specific genes and subtracting the average expression value of the TE-specific genes.

## Additional Information

**How to cite this article**: Nestorov, P. *et al.* Dynamic expression of chromatin modifiers during developmental transitions in mouse preimplantation embryos. *Sci. Rep.* **5**, 14347; doi: 10.1038/srep14347 (2015).

## Supplementary Material

Supplementary Information

Supplementary Dataset 1

Supplementary Dataset 2

Supplementary Dataset 3

Supplementary Dataset 4

Supplementary Dataset 5
