# Supplementary material for: Dynamic expression of chromatin modifiers during developmental transitions in mouse preimplantation embryos
Source: Sci Rep. 2015 Sep 25;5:14347. doi: 10.1038/srep14347 (PMC4585904; doi:10.1038/srep14347)
Supplement: Supplementary Information [file srep14347-s1.pdf]

Dynamic expression of chromatin modifiers during developmental transitions in mouse preimplantation embryos

Peter Nestorov [1,2,#], Hans-Rudolf Hotz [1] , Zichuan Liu [1] and Antoine H.F.M. Peters[1,2,*]

SUPPLEMENTARY INFORMATION

Supplementary Table 1 | Gene expression of 156 genes in mouse pre-implantation embryos.

Normalized log2 expression values for all genes and all samples. The "extended gene name" gives information on the suggested molecular function of a given gene (inferred from information in public databases, literature, as well as personal communication). It first shows the official gene name, followed by an alternative gene name (where applicable), the main molecular activity (CR – chromatin remodelling; DNA – DNA binding; DNAme – DNA methylation; HDM – histone demethylation; HR – histone binding; HMT – histone methylation; HK – housekeeping; SIG – signalling), important functional domains (SET – SET histone methyltransferase domain; ZnF – Zn-finger domain; ATPase; Helicase; Kinase; CD – chromodomain; JmjC/JmjD - Jumonji C/D domains) and finally the associated histone modification. In addition, the genes that were taken from the Guo *et al.* study[5], are highlighted in yellow, and the three reference genes are highlighted in blue.

Supplementary Table 2 | Mean expression values and PCA loading scores.

This table relates to Fig. 1f and gives the mean values for each gene in MII oocytes, 16-cell and 32-cell blastomeres, and all samples respectively. It also shows the PC loadings for each gene for the first two components in the PCA.

Supplementary Table 3 | Cell fate and lineage score of 16- and 32-cell embryos.

For each of the samples of the 16-cell and 32-cell embryos, a "Lineage Score" was calculated by taking the average expression value of the ICM genes highlighted in **Fig. 3a** and subtracting the average expression value for the highlighted trophectoderm genes. Samples with a resulting Lineage score lower than -3 were labelled as TE, and samples with a score higher than 4 were labelled as ICM. The colouring in **Fig. 3b** refers to this table.

Supplementary Table 4 | Correlation matrix of blastomeres of 16- and 32-cell embryos.

Spearman rank correlation matrix for all analysed genes based on the expression data from 16-cell and 32-cell blastomeres samples.

Supplementary Table 5 | p-values for correlation matrix of blastomeres of 16- and 32-cell embryos.

p-value matrix corresponding to the correlation matrix in Supp. Table 4.

a

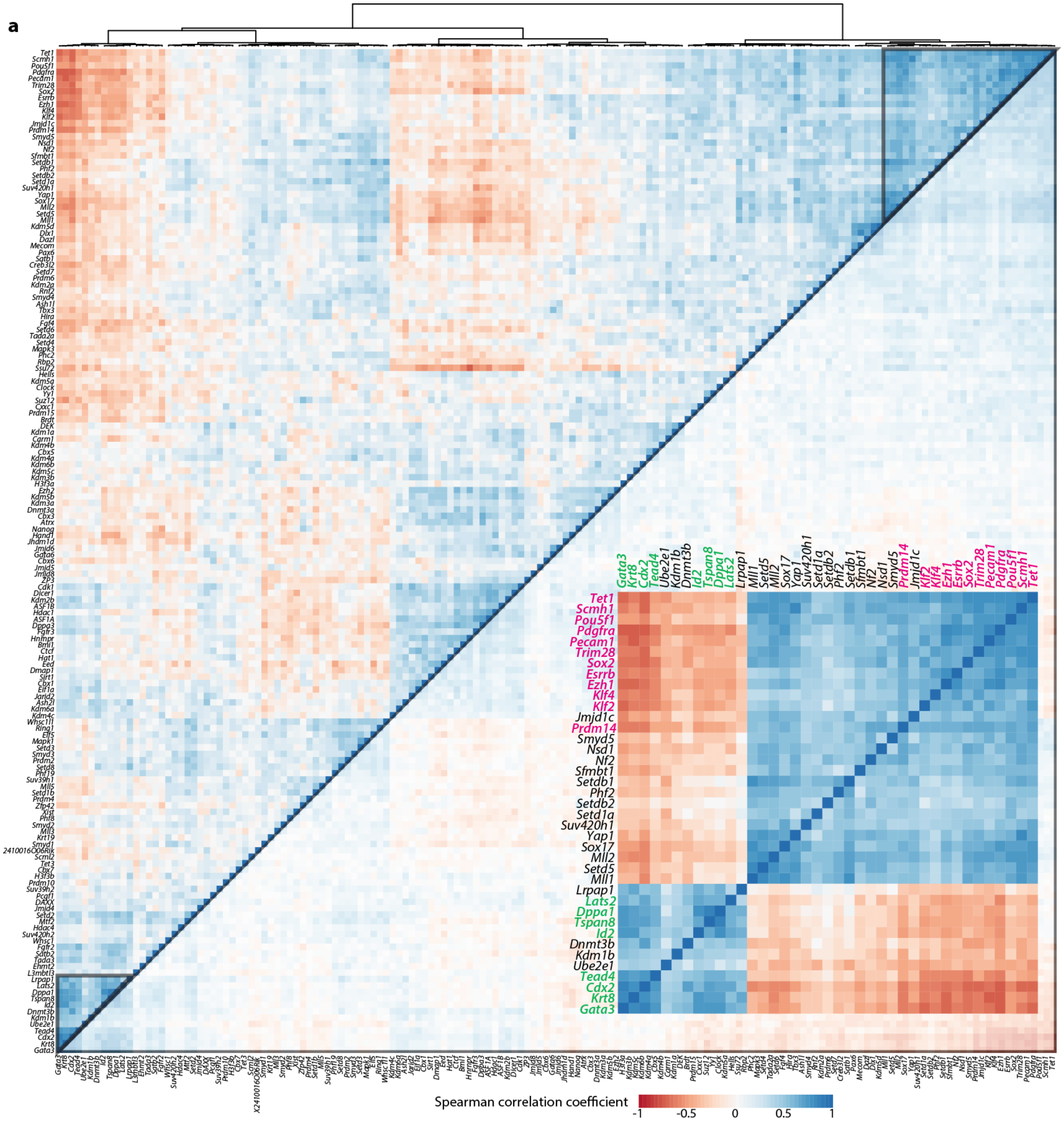
Gata3
Krt8
Cdx2
Tead4
Ube2e1
Kdm1b
Dnmt3b
Id2
Tspan8
Dppa1
Lats2
Lrpap1
Mll1
Setd5
Mll2
Sox17
Yap1
Suv420h1
Setd1a
Setdb2
Phf2
Setdb1
Sfmbt1
Nf2
Nsd1
Smyd5
Prdm14
Jmjd1c
Klf2
Klf4
Ezh1
Esrrb
Sox2
Trim28
Pecam1
Pdgfra
Pou5f1
Scmh1
Tet1
Spearman correlation coefficient
-1
-0.5
0
0.5
1

b

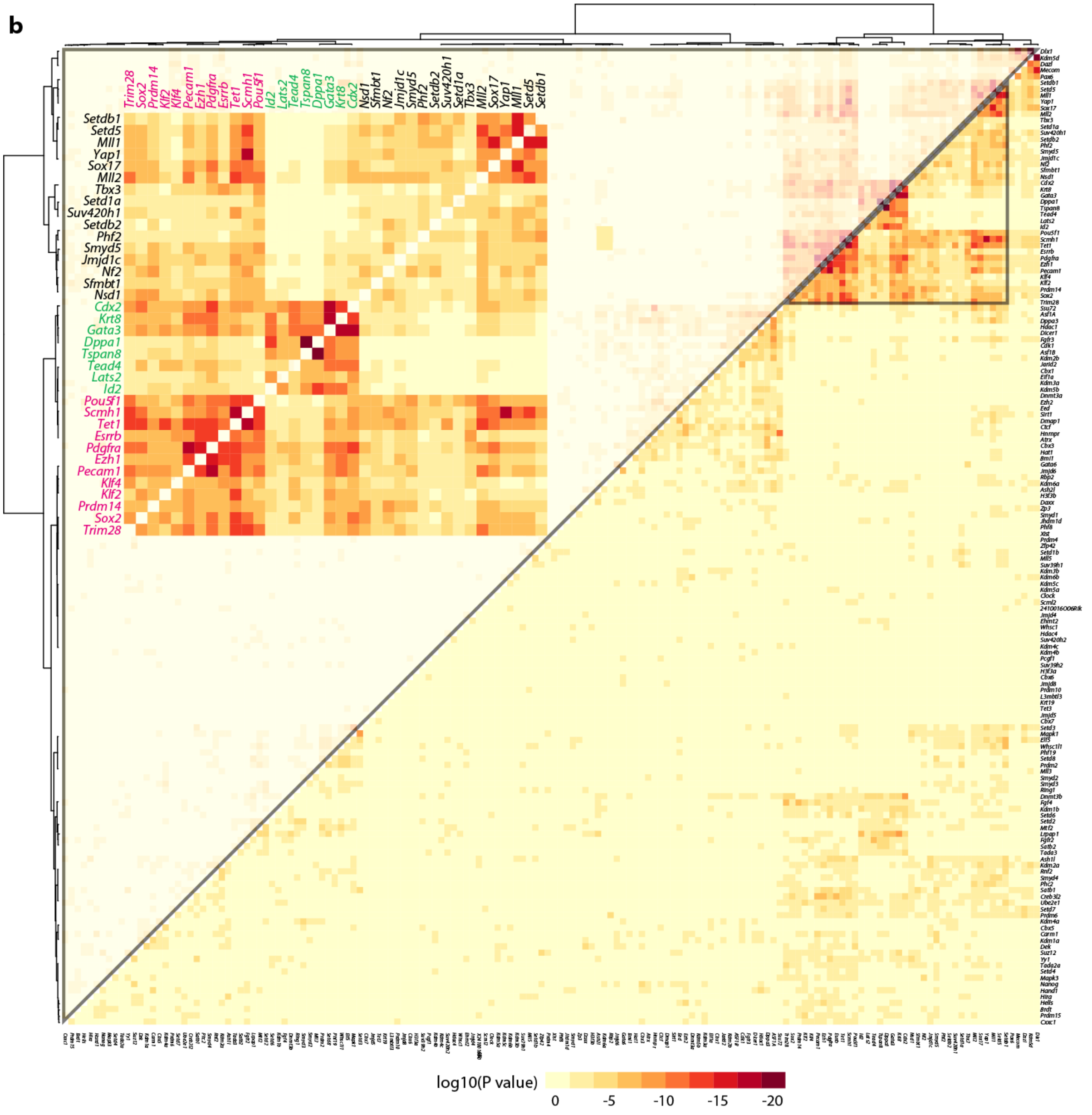

Supplementary Figure 1 | Correlation matrix for gene expression in 16- and 32-cell embryos.

a, Clustered correlation matrix for all genes based on expression data from 16-cell and 32-cell blastomeres samples. Lower right: zoom-in of highlighted clusters as in Fig. 3a.

b, Corresponding clustered p-value matrix. Upper-left: zoom-in of highlighted cluster, which comprises the lineage specific genes. Colouring of gene names corresponds to Fig. 3a, i.e. purple for ICM-specific genes and green for TE-specific genes.

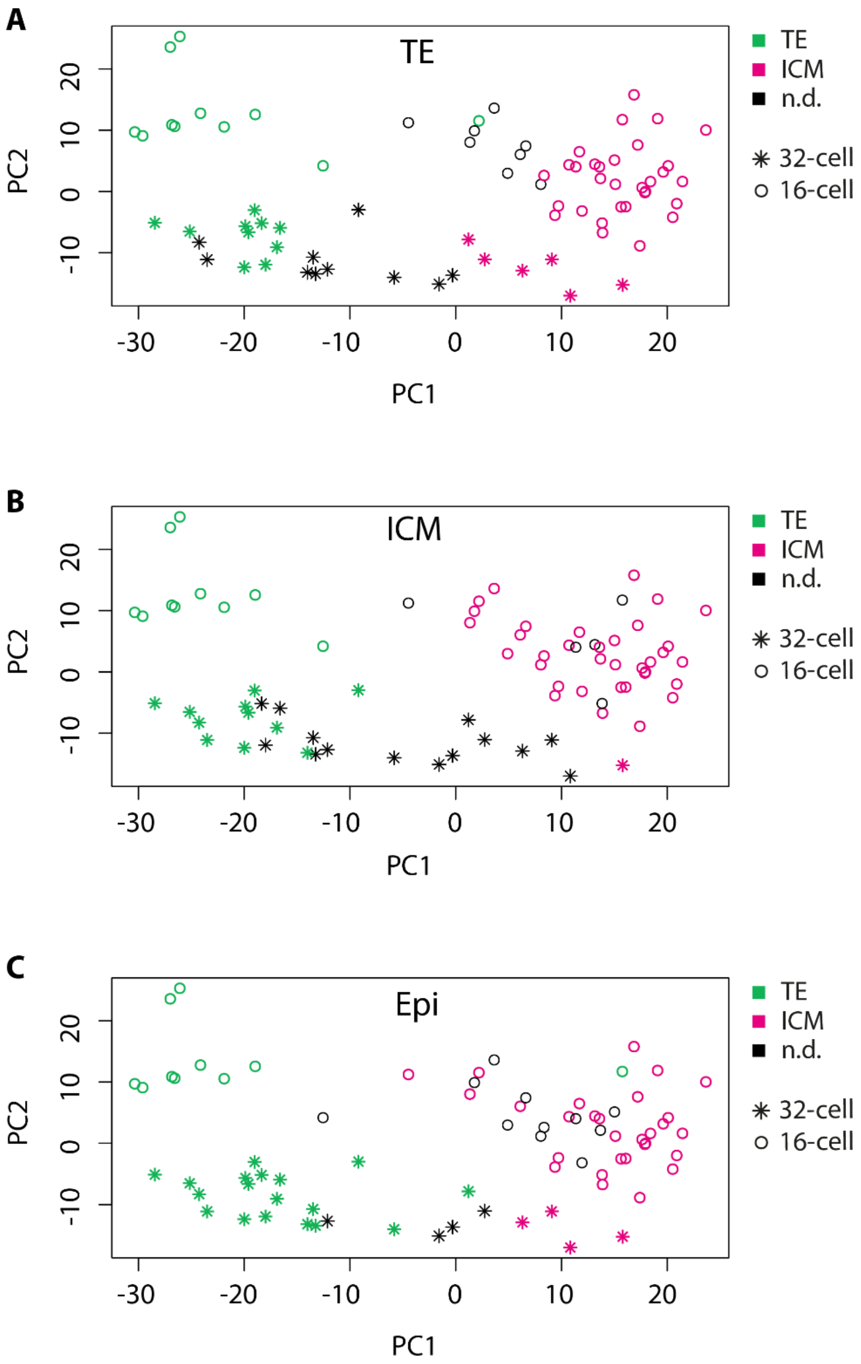

Supplementary Figure 2 | Cell fate assignment.

A PCA was done for 16- and 32-cell embryos (as in Fig. 3c) and a cell fate was assigned to each sample according to the expression levels (low, mid or high) of TE-specific genes (**a**), ICM-specific transcription factors and signalling molecule genes (**b**) or ICM-specific chromatin modifier genes (**c**) (see Supp. Table 3). The shape of the points indicates the stage, while the colour corresponds to the assigned cell fate.
